# Nonsuicidal Self-injury: A Systematic Review

**DOI:** 10.3389/fpsyg.2017.01946

**Published:** 2017-11-08

**Authors:** Annarosa Cipriano, Stefania Cella, Paolo Cotrufo

**Affiliations:** Observatory on Eating Disorders, Department of Psychology, University of Campania “Luigi Vanvitelli”, Caserta, Italy

**Keywords:** deliberate self-harm, self-injury, nonsuicidal self-injury, NSSI, DSM-5

## Abstract

**Objective:** Nonsuicidal self-injury (NSSI) refers to the intentional self-inflicted destruction of body tissue without suicidal intention and for purposes not socially sanctioned. Our paper presents an up-to-date overview on nonsuicidal, self-injurious behaviors.

**Method:** In accordance with PRISMA guidelines, a systematic literature search was conducted across two databases, PubMed and PsycARTICLES, regarding the main features of NSSI with a focus on epidemiological and etiologic data, diagnostic criteria, and functions. All English articles, published between 1998 and 2016, were considered, and screened against a priori inclusion/exclusion criteria. The search terms include: self-harm, self-injury, NSSI, epidemiology, comorbidity, gender, functions and DSM. We also examined the references of the retrieved articles.

**Results:** NSSI is most common among adolescents and young adults, and the age of onset is reported to occur between 12 and 14 years. Comorbidity with borderline personality disorder (BPD) and eating disorders is often reported. DSM-5 includes NSSI as a condition requiring further study. This review gives an overview of the prevalence rates (7.5–46.5% adolescents, 38.9% university students, 4–23% adults) and main causes that appear to stem from childhood trauma, comorbidity with many other disorders and several functions of NSSI, and the potential independence of a NSSI disorder.

**Conclusion:** Over the years, interest in NSSI grew to such an extent that an ongoing debate was instigated on whether NSSI should be considered as a diagnosis in its own right and given its own category. This paper provides an up-to-date overview on self-injury, what is known about it and what remains to be done. Clearly, our understanding of the main issues of NSSI has increased in last two decades. However, future researches is needed to examine the developmental trajectories, cultural backgrounds and shed light on the risk factors and functions as well as clarify its role as an independent diagnostic entity.

## Introduction

Nonsuicidal Self-Injury (NSSI) behavior is a growing clinical and public health problem. NSSI is defined as the direct and deliberate destruction of one's own bodily tissue in the absence of lethal intent and for reasons not socially sanctioned (Favazza, 1996; Nock, 2010). Common forms of NSSI include behaviors such as cutting, burning, scratching, and self-hitting (Briere and Gil, 1998; Laye-Gindhu and Schonert-Reichl, 2005; Whitlock et al., 2006; Klonsky and Muehlenkamp, 2007) and most self-injurers report using multiple method (Favazza and Conterio, 1988; Favazza, 1992). Evidences focused on the psychological intentions underlying NSSI demonstrated that the behavior serves a variety of function, both interpersonal and intrapersonal, that are not mutually exclusive (Suyemoto, 1998; Nock and Prinstein, 2004; Klonsky, 2007). Initial research on self-injurious behavior focused on studies in clinical settings (Pattison and Kahan, 1983), primarily with female subjects (Favazza and Conterio, 1989; Favazza et al., 1989; Herpertz, 1995; Suyemoto and MacDonald, 1995).

Epidemiological studies have endured due to the over-inclusive definition of behavior, with and without suicidal intent, as well as the dearth of consistent assessment measures. Earlier estimates ranged from 40 to 82% among adolescents in psychiatric inpatient settings (Darche, 1990; DiClemente et al., 1991) and stated that ~4% of the general population have a history of NSSI (Briere and Gil, 1998). Most recently researchers noticed that self-injurious behavior is more prevalent even among adolescents and young adults. The first attempt to describe this behavior can be seen in the book “*Man against himself*” by Menninger (1938), in which the author defined self-injurious behavior as a sort of “partial suicide.” There has been an absence of generally agreed upon terminology and, over the years, several different terms to define self-injurious behaviors have appeared in literature: syndrome of delicate self-cutting (Pao, 1969), deliberate self-harm (Pattison and Kahan, 1983), self-wounding (Tantam and Whittaker, 1992), moderate self-mutilation (Favazza and Rosenthal, 1993), self-mutilation (Ross and Heath, 2002); some of which include suicidal behaviors, risk taking, and an indirect form of self-harm (Favazza, 1996). The lack of consensus regarding terminology and definition has made the understanding of such behaviors very difficult. Self-injury is a common but—as yet—poorly understood phenomenon (Klonsky and Muehlenkamp, 2007).

Self-injury has long been linked to other disorders as well, including post-traumatic stress disorder (Briere and Gil, 1998; Bolognini et al., 2003), depressive disorders (Darche, 1990), obsessive-compulsive disorder (Bolognini et al., 2003), anxiety disorder (Darche, 1990; Simeon and Favazza, 2001), borderline personality disorder (BPD) (Klonsky et al., 2003; Nock et al., 2006), and eating disorder (Iannaccone et al., 2013). Many researchers and clinicians have argued for the adoption of a NSSI disorder: some of the earliest attempts to define such a syndrome being made by Graff and Mallin (1967); Pao (1969), and Rosenthal et al. (1972). Those first attempted failed (Favazza and Rosenthal, 1990) due to the inclusion of suicide attempts in the definitions. Kahan and Pattison (1984) differentiated self-harming behaviors from suicide and proposed a separate diagnostic disorder: the deliberate self-harm syndrome (DSH). Later, Favazza and Rosenthal (1990) suggested that habitual and repetitive self-injurious behavior could be considered as an impulse control disorder: the repetitive self-mutilation syndrome. Muehlenkamp (2005) also proposed that repetitive NSSI should be regarded as a separate diagnostic disorder. More recently, Wilkinson and Goodyer (2011) proposed that giving NSSI its own diagnostic category would improve communication and increase research on etiology, its treatment and outcome. There have been many arguments over NSSI, but given the high prevalence of self-injurious behaviors among clinical and community samples of adolescents (Muehlenkamp et al., 2012; Swannell et al., 2014), and associated clinical and functional impairment, the Childhood and Mood Disorders work-group of the DSM-5 proposed the inclusion of NSSI as a separate diagnostic disorder (Shaffer and Jacobson, 2009). Despite its criteria undergoing several revisions, due to a lack of research on the full set proposed criteria, inadequate sample size and unacceptably low inter-rater reliability results in the DSM-5 field trials (Regier et al., 2013), the NSSI disorder (NSSID) was only included as a condition requiring further study, in section 3 of DSM-5, and it represents an important step forward in recognizing NSSI as a disorder in its own right (Selby et al., 2015) and in promoting further research. Given the contrasts and conflicting data present in literature on NSSI, the aim of the present study is to systematize this broad field of research, focusing on (1) proposed diagnostic criteria for the DSM-5, (2) epidemiology, (3) comorbidity, (4) etiology, and (5) functions.

## Materials and methods

### Data source and search strategy

The present study followed the Preferred Reporting Items for Systematic Reviews and Meta-Analysis (PRISMA, Liberati et al., 2009). PubMed and PsycARTICLES databases were searched for eligible studies published in English between 1998 and 2016. The following combinations of search terms were employed: 1. *self-harm* OR, *self-injury* OR, *nonsuicidal self-injury* OR, *NSSI*, 2. epidemiology, 3. comorbidity, 4. gender, 5. Functions, 6. DSM. Additionally, we also examined the references of the articles identified in the search.

### Study selection

**Figure 1:**
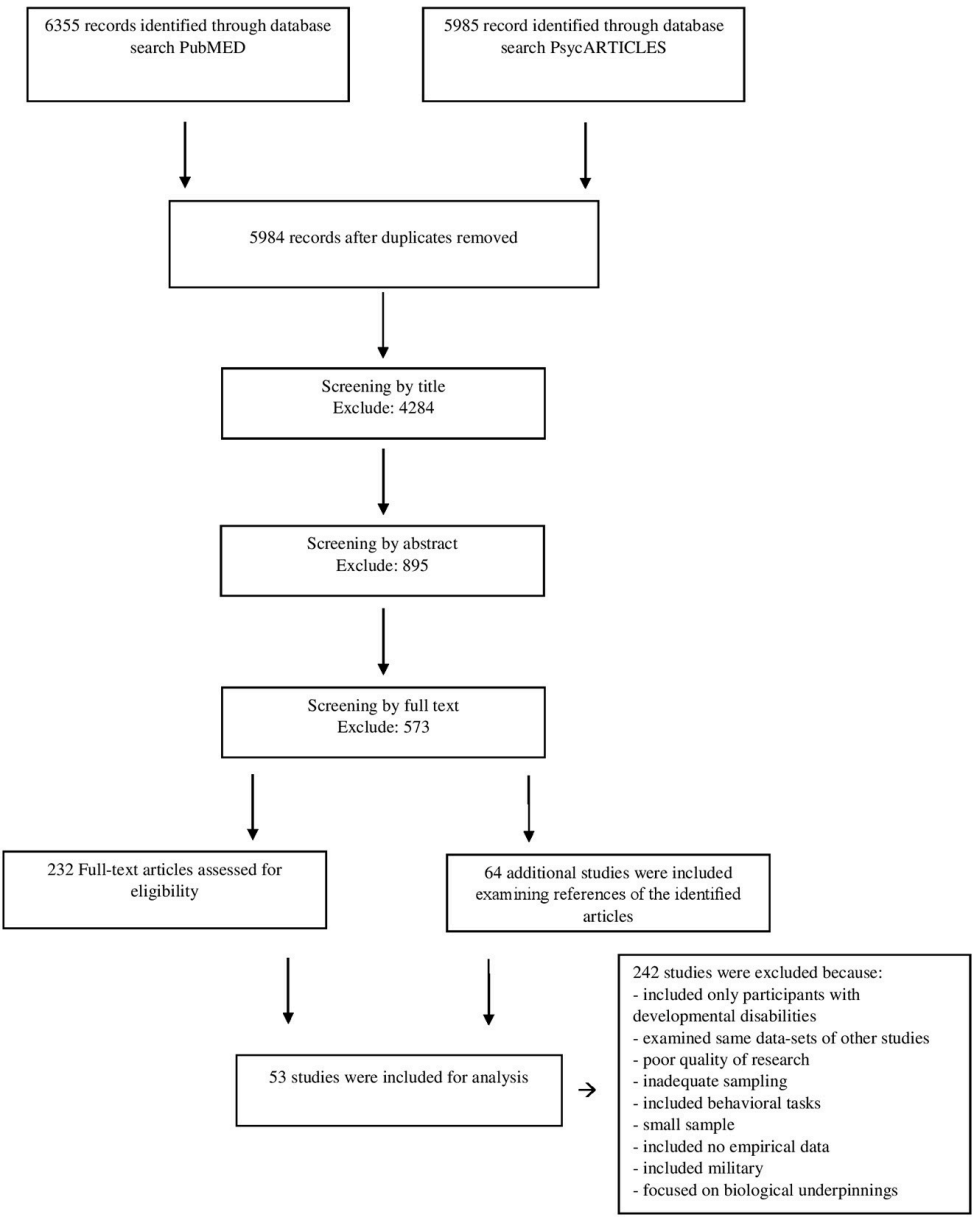
PRISMA flow diagram of study selection.

Figure 1 shows the selection of included studies. In total, the initial database search yielded 12340 abstract, of which 6356 duplicate were removed. Articles were first screened by title and abstract by two independent reviewers. Of the remaining studies, the full text was obtained and inspected independently by the same two authors to ensure that the inclusion/exclusion criteria were met.

### Inclusion/exclusion criteria

To be included in this review, studies had to: (a) be published between 1998 and 2016, (b) reported empirical data, (c) provide a definition of self-injury and of their method of assessment, (d) not focus on treatment for NSSI, (e) be published in the English language. There were no restrictions on participant.

Reason for exclusion were: (a) samples could be not categorized as universal, (b) studies were based on the same results already found in another publication, (c) the full text was not available.

## Results

We identified 53 studies that met the inclusion criteria for this review. Table 1 provide a summary of the data obtained from each study.

**Table 1:** Study characteristics.

**References**	**Sample**	**Sample size (female %)**	**Mean age**	**Assessing tools**	**Prevalence**	**Endorsed criteria (%)**
**DIAGNOSTIC CRITERIA**						
Andover, 2014	Community	548 (46.5)	35.70 (*SD* = 12.23)	FASM–Self report questionnaire developed on DSM-5 proposed criteria	23^c^	A: 20.8 B1: 60.8 B2: 8.8 B3: 26 C1: 82.4 C2: 37.6 C3: 19.4 E: Distress 8.08–Impairment 60.8
Barrocas et al., 2012	School	665 (55)	11.6 (*SD* = 2.4)	SITBI–FASM	8% (1.5% NSSI disorder)^a^	A: 1.5 B4: 1.5 C: 1.5
Glenn and Klonsky, 2013	Psychiatric inpatient and partial hospitalization	198 (74)	15.13 (*SD* = 1.38)	ISAS	50% (78% of the self-injuring sample)^a^	A: 50 B1: 98
Gratz et al., 2015	Community	107 (80)	23.86 (*SD* = 4.87)	CANDI	37^c^	A: 77 B: 79 C: 81 D: 91 E: 41 F: 80
In-Albon et al., 2013	Psychiatric Inpatient	73 (100)	13–18 years	DSM-5 criteria reformulated as questions in a clinical interview	56.2^b^	A: 20.8 B1: 97.4 B2: 46.2 B3: 89.7 B4: 87.2 C: Distress 100–Impairment 69.2
Washburn et al., 2015	Clinical inpatients, partial hospitalization and intensive outpatients	511 (90)	17.3 (*SD* = 6.2)	ABASI	74^c^	A: 85.5 B1: 82 B2: 57.1 B3: 34.8 C1:91.3 C2: 72.8 C3: 71.6 E: 98.2 F: 98.2
Zetterqvist et al., 2013	Community students	3,060 (48.8)	15–17 years	FASM SITBI-SF-SR	6.7% (18.8% of NSSI sample)^b^	A: 85.5 B1: 98.5 B2: 73.2 B3: 37.3 B4: 99.5 C: Distress 76.8–Impairment 92.2
**References**	**Sample**	**Sample size (female%)**	**Mean age**	**Assessing tools**	**Prevalence (%)**	**Country**
**EPIDEMIOLOGY**						
Andover, 2014	Community	548 (46.5)	35.70 (*SD* = 12.23)	FASM–Self report questionnaire developed on DSM-5 proposed criteria	23^c^	USA
Barrocas et al., 2012	School	665 (55)	11.6 (*SD* = 2.4)	SITBI–FASM	8 (1.5 NSSI disorder)^a^	USA
Briere and Gil, 1998	Community Clinical Self-mutilative	927 (50) 390 (52) 93 (96)	46 (*SD* = 17) 36 (*SD* = 10) 35 (*SD* = 9)	Self-administered questionnaire	4.0 21.0 100	USA
Cerutti et al., 2011	School	234 (49.1)	16.47 (*SD* = 1.7)	DSHI–Italian Version	41.9	Italy
Cerutti et al., 2012	College	365 (62.79)	23.34 (*SD* = 4.06)	DSHI	38.9	Italy
Claes et al., 2007	Psychiatric inpatients	399 (66.4)	30.8 (*SD* = 12.2)	SIQ SHI	41.04	Belgium
Gratz et al., 2002	College	133 (67)	22.73 (SD = 6.17)	DSHI	38	USA
Gratz, 2006	College	249 (100)	23.29 (*SD* = 5.96)	DSHI	37	USA
Hilt et al., 2008a	School	508 (51)	–^*^	Self-report Questionnaire	7.5	USA
Kaess et al., 2013	Psychiatric inpatients	125 (50.4)	17.1 (*SD* = 3.1)	FASM	60	Germany
Kirchner et al., 2011	School	1171 (55.8)	3.96 (*SD* = 1.32)	YSR Spanish version	11.4	Spain
Klonsky, 2011	Community	439 (61)	55.5 (*SD* = 16.6)	Structured interview	5.9	USA (Exclude Alaska and Hawaii)
Kuentzel et al., 2012	College	5,680 (70.12)	22.2 (*SD* = 6.35)	Self-report questionnaire	12.8	USA
Laye-Gindhu and Schonert-Reichl, 2005	Community	424 (55.6)	15.34 (*SD* = 1.06)	Self-Harm questionnaire	15	Canada
Lloyd-Richardson et al., 2007	Community	633 (57)	15.5 (*SD* = 1.18)	FASM	46.5	USA
Muehlenkamp and Gutierrez, 2007	School	540 (62.3)	15.53 (*SD* = 1.42)	SHBQ	23.2	USA
Muehlenkamp et al., 2013	College	1,243 (59.8)	21.52 (*SD* = 4.15)	NSSI-AT	14.72	USA
Nock et al., 2006	Psychiatric inpatients	89 (74.15)	14.7 (*SD* = 1.4)	FASM	100	USA
Plener et al., 2009	School	665 (57.1)	14.8 (*SD* = 0.66)	SHBQ–OSI	26	Germany
Ross and Heath, 2002	School	440 (50.2)	–^*^	Semi structured Interview	14.8	Canada
Sornberger et al., 2012	School	7,126 (50.8)	14.92 (*SD* = 1.61)	Self-administered questionnaire	24.5	USA
Whitlock et al., 2006	College	2,875 (56.3)	18–24 years	Self-report questionnaire	17	USA
Whitlock et al., 2011	College	11,529 (57.6)	Under 25 years	Self-report questionnaire	15.3	USA
Yates et al., 2008	Community	155 (51.61)	26 years	SIBQ	16.8	USA
Zoroglu et al., 2003	School	839 (61.1)	15.9 (*SD* = 1.8)	Self-report questionnaire	21.4	Turkey
**References**	**Sample**	**Sample size (female %)**	**Mean age**	**Assessing tools**	**Prevalence (%)**	**Comorbidity**
**NSSI AND OTHER DISORDERS**						
Briere and Gil, 1998	Community Clinical Self-mutilative	927 (50) 390 (52) 93 (96)	46 (*SD* = 17) 36 (*SD* = 10) 35 (*SD* = 9)	Self-administered questionnaire	4.0 21.0 100	Post-traumatic stress disorder, unspecified dissociative disorder, borderline personality disorder and dissociative identity disorder, dissociation and depression
Cerutti et al., 2012	College	365 (62.79)	23.34 (*SD* = 4.06)	DSHI	38.9	Dissociation, depersonalization, and borderline personality symptoms
Claes et al., 2001	Psychiatric inpatients	134 (100)	Mean age	SIQ	44.6	Eating disorders, anxiety disorder and depression
Claes et al., 2007	Psychiatric inpatients	399 (66.4)	30.8 (*SD* = 12.2)	SIQ SHI	41.04	Personality disorders, depressive disorder, and obsessive-compulsive disorder
Eichen et al., 2016	College	508 (100)	20.61 (*SD* = 1.97)	FASM	13.8	Eating disorders, depressive disorder, anxiety disorder and difficulties with emotion regulation
Giletta et al., 2012	School	1,862 (49)	15.69 (*SD* = 0.87)	Self-report questionnaire	24	Depressive symptoms and substance use
Glenn and Klonsky, 2013	Psychiatric inpatient and partial hospitalization	198 (74)	15.13 (*SD* = 1.38)	ISAS	50% (78% of the self-injuring sample)^a^	Alcohol/substance use disorder, anxiety disorder, mood disorder, ADHD/disruptive behavior disorder, bulimia, borderline personality disorder and emotion dysregulation
Gratz et al., 2015	Community	107 (80)	23.86 (*SD* = 4.87)	CANDI	37^c^	Emotion dysregulation, borderline personality disorder, mood disorder, anxiety disorder, substance use disorder.
Hilt et al., 2008a	School	508 (51)	–^*^	Self-report Questionnaire	7.5	Maladaptive eating Habits and substance use
Iannaccone et al., 2013	Psychiatric inpatients and outpatients	65 (100)	27.46 (*SD* = 8.29)	Self-report questionnaire	50.9	Eating disorders, impulsivity, anxiety and depression
In-Albon et al., 2013	Psychiatric Inpatient	73 (100)	13–18 years	DSM-5 criteria reformulated as questions in a clinical interview	56.2^b^	Mood disorders, post-traumatic stress disorder, borderline personality disorder, anxiety disorders, oppositional deviant disorder, and bulimia nervosa
Jenkins et al., 2015	Clinical and control group	1,097 (53.6)	35.1 (*SD* = 10.3)	DSHI	18	Intermittent explosive disorder, personality disorders, mood and anxiety disorders, eating disorders, substance use disorder
Muehlenkamp and Gutierrez, 2007	School	540 (62.3)	15.53 (*SD* = 1.42)	SHBQ	23.2	Depressive symptoms
Nock et al., 2006	Psychiatric inpatients	89 (74.15)	14.7 (*SD* = 1.4)	FASM	100	Major depressive disorder, post-traumatic stress disorder, anxiety disorder, conduct and oppositional defiant disorder, substance abuse disorders and personality disorders (borderline, avoidant and paranoid personality disorders were most common)
Plener et al., 2009	School	665 (57.1)	14.8 years (*SD* = 0.66)	SHBQ–OSI	26	Depressive symptoms
Ross and Heath, 2002	School	440 (50.2)	–^*^	Semi structured Interview	14.8	Anxiety and depressive symptomatology
Selby et al., 2012	Clinical outpatients	571 (53)	Adults	Chart data	11.4^a^	Mood disorders, anxiety disorder, and Cluster A personality disorders
Turner et al., 2015	NSSI sample	100 (90)	31.57 (*SD* = 10.13)	DSHI	100	Mood and anxiety disorders, substance use disorders, eating disorders, psychotic disorders, and personality disorders.
**References**	**Sample**	**Sample size (female %)**	**Type of sample**	**Assessing tools**	**Prevalence (%)**	**Key findings**
**ETIOLOGY**						
Arens et al., 2012	College	407 (65)	20.33 (*SD* = 1.39)	DSHI	20	Childhood maltreatment
Auerbach et al., 2014	Clinical	194 (74.22)	15.53 (*SD* = 1.34)	SITBI	80.92	Child abuse
Briere and Gil, 1998	Community Clinical Self-mutilative	927 (50) 390 (52) 93 (96)	46 (*SD* = 17) 36 (*SD* = 10) 35 (*SD* = 9)	Self-administered questionnaire	4.0 21.0 100	Childhood trauma
Cerutti et al., 2011	School	234 (49.1)	16.47 (*SD* = 1.7)	DSHI–Italian Version	41.9	Dissociation and stress full life events
Goldstein et al., 2009	College	319 (65)	18.89 (*SD* = 2.30)	Self-report questionnaire	29.5	Depressive symptoms, physical abuse, emotional abuse, openness, sensation seeking and substance use
Gratz et al., 2002	College	133 (67)	22.73 (*SD* = 6.17)	DSHI	38	Dissociation, insecure paternal attachment, parental emotional neglect, childhood sexual abuse and childhood separation
Gratz, 2006	College	249 (100)	23.29 (*SD* = 5.96)	DSHI	37	Childhood maltreatment, low positive affect intensity/reactivity and emotional inexpressivity
Gratz and Chapman, 2007	College	97 (0)	22.67 (*SD* = 5.00)	DSHI	44	Physical abuse and emotion dysregulation
Jacobson et al., 2015	College	427 (73.3)	20.5 (*SD* = 4.5)	FASM	6	Emotional expressiveness
Kaess et al., 2013	Psychiatric inpatients	125 (50.4)	17.1 (*SD* = 3.1)	FASM	60	Adverse childhood experiences
Paivio and McCulloch, 2004	College	100 (100)	21 (*SD* = 1.66)	SIBQ	41	Child maltreatment
Tang et al., 2016	School	4,405 (49.67)	14.7 (*SD* = 1.9)	FASM	29.2	Stress full life events
Wan et al., 2015	School	14,211 (52.8)	5.1 (*SD* = 1.9)	FASM	24.9	Childhood abuse
Whitlock et al., 2006	College	2,875 (56.3)	18–24 years	Self-report questionnaire	17	Emotional, physical and sexual abuse
Yates et al., 2008	Community	155 (51.61)	26	SIBQ	16.8	Child sexual and physical abuse
Zoroglu et al., 2003	School	839 (61.1)	15.9 (*SD* = 1.8)	Self-report questionnaire	21.4	Physical, emotional and sexual abuse, neglect and dissociation
**References**	**Sample**	**Sample size (female %)**	**Mean age**	**Assessing tools**	**Prevalence**	**Functions**
**FUNCTIONS**						
Andover, 2014	Community	548 (46.5)	35.70 (*SD* = 12.23)	FASM–Self report questionnaire developed on DSM-5 proposed criteria	23^c^	Automatic positive, automatic negative, social negative and social positive
Calvete et al., 2015	School	1,864 (51.45)	15.3 (*SD* = 1.97)	FASM	55.6	Automatic positive, automatic negative, social negative and social positive
Claes et al., 2007	Psychiatric inpatients	399 (66.4)	30.8 (*SD* = 12.2)	SIQ SHI	41.04	Automatic and social
Giletta et al., 2012	School	1862 (49)	15.69 (*SD* = 0.87)	Self-report questionnaire	24	Internal and interpersonal
Glenn and Klonsky, 2013	Psychiatric inpatient and partial hospitalization	198 (74)	15.13 (*SD* = 1.38)	ISAS	50%^a^ (78% of the self-injuring sample)	Affect regulation, marking distress, self-punishment and anti-dissociation
Hilt et al., 2008b	Community	94 (100)	10–15 years	FASM	56.4	Automatic positive, automatic negative, social negative and social positive
Kaess et al., 2013	Psychiatric inpatients	125 (50.4)	17.1 (*SD* = 3.1)	FASM	60	Automatic, interpersonal and peer identification
Klonsky, 2011	Community	439 (61)	55.5 (*SD* = 16.6)	Structured interview	5.9	Affect regulation, self-punishment and interpersonal
Laye-Gindhu and Schonert-Reichl, 2005	Community	424 (55.6)	15.34 (*SD* = 1.06)	Self-Harm questionnaire	15	Affect regulation, self-punishment, distraction from problems, communicate with or influence others
Lloyd-Richardson et al., 2007	Community	633 (57)	15.5 years (*SD* = 1.18)	FASM	46.5	Automatic positive, automatic negative, social negative and social positive
Muehlenkamp et al., 2013	College	1,243 (59.8)	21.52 (*SD* = 4.15)	NSSI-AT	14.72	Emotional regulation and social
Nock and Prinstein, 2004	Psychiatric Inpatients	108 (70.37)	14.8 (*SD* = 1.4)	FASM	82.4	Automatic positive, automatic negative, social negative and social positive
Turner et al., 2012	NSSI sample	162 (100)	22.47 years (*SD* = 7.14)	QNSII–SASII	100	Emotion relief, feeling generation, self-punishment-, interpersonal communication, interpersonal influence
Turner et al., 2016	NSSI sample	60 (85)	23.25 (*SD* = 4.25)	DSHI	100	Affect regulation and interpersonal
Zetterqvist et al., 2013	Community students	3,060 (48.8)	15–17 years	FASM SITBI-SF-SR	6.7% (18.8% of NSSI sample)^b^	Automatic positive, automatic negative, social negative and social positive

*Study by Lengel and Mullins-Sweatt (2013), on expert ratings, and meta-analysis by Bresin and Schoenleber were not included NSSI criteria used*.

a *Shaffer and Jacobson (2009)*.

b *American Psychiatric Association (2012)*.

c *DSM-5 (American Psychiatric Association, 2013)*.

* *Data not reported. Alexian Brothers Assessment of Self-Injury (ABASI); Clinician-Administered Nonsuicidal Self-Injury Disorder Index (CANDI); Deliberate Self Harm Inventory (DSHI); Inventory of Statements About Self-Injury (ISAS); Functional Assessment of Self-Mutilation (FASM); Nonsuicidal Self-Injury-Assessment Tool (NSSI-AT); Ottawa Self-Injury Inventory (OSI); Questionnaire for Nonsuicidal Self-Injury (QNSSI); Self-Harm Behavior Questionnaire (SHBQ); Self-Injurious Behavior Questionnaire (SIBQ); Self-Injurious Thoughts and Behaviors Interview-Short Form-Self-Report (SITBI-SF-SR); Self-Injurious Thoughts and Behaviors Interview (SITBI); Self-Injury Questionnaire (SIQ); Self-Harm Inventory (SHI); Suicidal Attempt Self-Injury Interview (SASII); Youth Self Report (YSR)*.

### Diagnostic criteria

Recent research on NSSI disorder (American Psychiatric Association, 2013) found that a high percentage of those who self-injure met the DSM-5 proposed criteria (Glenn and Klonsky, 2013; Washburn et al., 2015). In a community sample of 3,097 Swedish adolescents, Zetterqvist et al. (2013) found that 6.7% met the criteria, whereas in a sample of adolescent impatients prevalence was 50% (Glenn and Klonsky, 2013). Empirical data on a potential NSSI disorder have collected among clinical and community samples of adolescents and adults, using different version of proposed criteria (Shaffer and Jacobson, 2009; American Psychiatric Association, 2012, 2013). In a combination samples of inpatient and intensive outpatient subjects 85.5% met Criterion A (Washburn et al., 2015). Two hundred and five of adolescents reported frequent and multiple forms of NSSI (Zetterqvist et al., 2013). For Criterion B, high endorsement was found in clinical sample of adolescents and adults (In-Albon et al., 2013; Zetterqvist et al., 2013; Washburn et al., 2015), as well as in general adult samples (Andover, 2014; Gratz et al., 2015). Almost all of adolescents (99.5%) who fulfilled criteria for NSSI disorder reported engaging NSSI to relieve both intrapersonal and interpersonal difficulties (Zetterqvist et al., 2013), likewise most patients engaged in NSSI with the expectation to lead relief from either a negative feeling or cognitive state (82.0%) or resolution of an interpersonal problem (57.1%) (Washburn et al., 2015). Automatic functions are reported significantly more often than social functions, in adolescents and adults (Zetterqvist et al., 2013; Andover, 2014). In Washburn et al. (2015), it was also rare to meet Criterion B without also meeting Criterion C: C1 (American Psychiatric Association, 2013) was the most commonly endorsed symptom, but patients also reported high endorsement (62.4%) for all three symptoms.

Clinicians and expert NSSI researchers described experiencing negative feeling or through prior to the NSSI behavior as a prototypic symptom, following by preoccupation and urge to engage with a less agreement (Lengel and Mullins-Sweatt, 2013). Criterion D—behavior act for purposes not socially sanctioned—had an agreement of 88% as being relevant characteristic to the disorder (Lengel and Mullins-Sweatt, 2013). The presence of clinically significant distress or impairment (Criterion E) is considered difficult to assess, NSSI behavior would lead relief rather than impairing. NSSID group reported more distress and impairment in functioning than non-NSSID group (Zetterqvist et al., 2013; Andover, 2014; Gratz et al., 2015). Several studies have assessed Criterion F using indirect methods (e.g., In-Albon et al., 2013; Andover, 2014).

### Epidemiology

Prevalence rates of NSSI in adolescents fall between 7.5 and 46.5%, rising to 38.9% among university students and 4–23% among adults (Briere and Gil, 1998; Gratz et al., 2002; Whitlock et al., 2006; Lloyd-Richardson et al., 2007; Hilt et al., 2008a; Plener et al., 2009; Cerutti et al., 2012; Andover, 2014). Although self-injurious behavior is a widespread phenomenon, data vary considerably across samples. The age onset of NSSI most often occurs in early adolescence, between 12 and 14 years (Nock et al., 2006; Muehlenkamp and Gutierrez, 2007; Cerutti et al., 2011), but findings have also reported NSSI behavior in children under the age of 12 (Barrocas et al., 2012). The most common method was self-cutting (over 70%) followed by head banging, scratching, hitting and burning (Briere and Gil, 1998; Laye-Gindhu and Schonert-Reichl, 2005; Gratz, 2006; Whitlock et al., 2006). However, most individuals who engage in NSSI employ more than one method (e.g., Whitlock et al., 2011) acting on the arms, legs, wrists and stomach (Whitlock et al., 2006; Lloyd-Richardson et al., 2007; Klonsky, 2011; Sornberger et al., 2012). The results from some studies suggested that women displayed more NSSI behaviors than males, in both clinical and non-clinical samples (Ross and Heath, 2002; Laye-Gindhu and Schonert-Reichl, 2005; Whitlock et al., 2006, 2011; Claes et al., 2007; Muehlenkamp and Gutierrez, 2007; Yates et al., 2008; Plener et al., 2009; Sornberger et al., 2012; Muehlenkamp et al., 2013). A meta-analysis by Bresin and Schoenleber (2015) demonstrated that women are slightly more likely than men to engage in NSSI.

Differences concern also the type of method chosen: self-cutting is most common among women, that were more likely than men to engage in methods of NSSI that generally involve blood (Sornberger et al., 2012), whereas hitting, burning and banging are most common among men (Laye-Gindhu and Schonert-Reichl, 2005; Claes et al., 2007). On the other hand, equal rates of NSSI between the genders have been reported within samples of adolescents, college students, and adults (Briere and Gil, 1998; Nock et al., 2006; Lloyd-Richardson et al., 2007; Hilt et al., 2008a; Cerutti et al., 2011, 2012; Kirchner et al., 2011; Kaess et al., 2013), as well as clinical samples of adults (Briere and Gil, 1998). Although no race differences were noted in NSSI rate among adolescents and university samples (Gratz et al., 2002; Hilt et al., 2008a), data on ethnic/minority groups are scarce. Within ethnically diverse sample, multiracial college students reported high prevalence rates (20.8%), followed by Caucasian (16.8) and Hispanic (17%) (Kuentzel et al., 2012). However, research on non-Caucasian subjects was limited to few countries. Among Chinese students prevalence rates of NSSI ranged 24.9–29.2% (Wan et al., 2015; Tang et al., 2016), likewise Zoroglu et al. (2003) reported that 21.4% of Turkish adolescents engage NSSI.

### NSSI and other disorders

According to research literature, NSSI is often associated with several maladaptive outcomes. Most notably, there is an association between NSSI and the diagnosis of BPD (Briere and Gil, 1998; Nock et al., 2006; Glenn and Klonsky, 2013; Gratz et al., 2015). Although listed as a diagnostic criterion for BPD (DSM-5, American Psychiatric Association, 2013), NSSI may also occur in individuals who do not receive BPD diagnosis, and not every individual who receives BPD diagnosis engages in self-harm behaviors (e.g., In-Albon et al., 2013). Differences between NSSI-group and BPD-group would suggest to define NSSI as syndrome in its own right (Selby et al., 2012; Turner et al., 2015). Even though NSSI and suicidal behavior are distinct, suicide attempts and suicide ideation were found in both clinical and non-clinical samples of adolescents (Nock et al., 2006; Plener et al., 2009).

Exploring the association between NSSI and psychiatric diagnoses, several researchers have reported self-injurious behavior in a wide range of other disorders, such as post-traumatic stress disorder (PTSD), dissociative disorder, conduct disorder, obsessive-compulsive disorder, intermittent explosive disorder, anxiety and mood disorder, substance use disorder, bulimia, and dissociative identity disorder (Briere and Gil, 1998; Nock et al., 2006; Claes et al., 2007; Selby et al., 2012; Glenn and Klonsky, 2013; In-Albon et al., 2013; Gratz et al., 2015; Jenkins et al., 2015; Turner et al., 2015). Furthermore, in a study on DSH behavior among young Italian adults, individuals with a history of DSH, compared with individuals with no history of DSH, reported higher levels of dissociations and depersonalization (Cerutti et al., 2012). In addition, a relationship between NSSI and eating disorders often appears (Claes et al., 2001; Iannaccone et al., 2013; Eichen et al., 2016), although not all researchers confirm such an association (Selby et al., 2012). Cerutti et al. (2012) found that adults with NSSI history reported negative attitudes toward the body and lower levels of body protection. In both clinical and non-clinical samples, those who self-injury were more likely to report depressive symptomatology and anxiety (Ross and Heath, 2002; Muehlenkamp and Gutierrez, 2007; Giletta et al., 2012; Selby et al., 2012). Moreover, results provided significantly higher rates of both internalizing (Nock et al., 2006; Glenn and Klonsky, 2013; In-Albon et al., 2013) and externalizing disorders (Nock et al., 2006). Adolescents who engage in NSSI were more likely to present several health-risk behaviors, such as substance abuse, risky sexual behaviors, and maladaptive eating habits (Hilt et al., 2008a; Giletta et al., 2012). In a study that assess potential NSSI disorder (Gratz et al., 2015) participants who met the proposed criteria for NSSID (DSM-5, American Psychiatric Association, 2013) differed from NSSI-group and reported significantly more depression, anxiety and stress symptoms, and BPD disorder.

### Etiology

The potential etiologic factors of NSSI may be divided into two major categories: individual (e.g., emotional dysregulation, psychiatric disorders) and environmental (e.g., childhood maltreatments, attachment disruption). Most research focused on early childhood traumatic experiences found that childhood maltreatments emerged as a predictor of NSSI within adolescents and college students (Paivio and McCulloch, 2004; Gratz, 2006; Arens et al., 2012; Auerbach et al., 2014; Wan et al., 2015). Exploration of environmental contributors revealed that childhood sexual abuse would present a strong link with NSSI development (Briere and Gil, 1998; Gratz et al., 2002; Gratz, 2006; Gratz and Chapman, 2007; Yates et al., 2008; Auerbach et al., 2014). However, other researchers have not found a strong association with sexual abuse (Zoroglu et al., 2003; Whitlock et al., 2006; Goldstein et al., 2009). In their study among college female students, Gratz (2006) found that both environmental and individual factors were strongly associated with NSSI, as well their interaction. Gratz et al. (2002) emphasized the role of parental relationship in the etiology of self-injurious behaviors: insecure paternal attachment and both maternal and paternal emotional neglect were significant predictors of NSSI within women, whereas NSSI in men was primarily predicted by childhood separation (usually from father). Furthermore, maternal rejection appeared the only significant predictor among psychiatric inpatients sample (Kaess et al., 2013).

Most recent studies have investigated the role of stress-full life events in the etiology of NSSI (Cerutti et al., 2011; Tang et al., 2016). Among Chinese adolescents, Tang et al. (2016) found that adverse life experiences were associated with moderate and severe NSSI and a lesser risk of engaging NSSI in those who had a good emotional regulation. Examining individual factors, results reported that NSSI frequency was strongly predicted by emotion dysregulation and affect intensity/reactivity within men (Gratz and Chapman, 2007), and by emotional inexpressivity within women (Gratz, 2006). Low emotional expressiveness would have a role in engagement in NSSI (Jacobson et al., 2015). Results of a regression analysis showed that difficulties to identify and express emotional experience appropriately (i.e., alexithymia) mediated the relation between childhood trauma (except sexual abuse) and NSSI (Paivio and McCulloch, 2004).

### Functions

Engage in NSSI may serves several functions that are not mutually exclusive (Nock and Prinstein, 2004; Klonsky, 2011). The most common function seems to be the affect regulation (Nock and Prinstein, 2004; Laye-Gindhu and Schonert-Reichl, 2005; Claes et al., 2007; Muehlenkamp et al., 2013). Indeed, negative emotions, such as anger, anxiety, depression, and loneliness, tend to occur before the NSSI behavior, whereas an increase in positive emotions and a decrease in negative emotions would follow as well (e.g., Laye-Gindhu and Schonert-Reichl, 2005; Claes et al., 2007). Moreover, Giletta et al. (2012) found that engaging NSSI were strongly associated with depressive feelings among Italian, USA and Dutch samples. NSSI may represent a strategy for affect regulation (Laye-Gindhu and Schonert-Reichl, 2005). Nock and Prinstein (2004) proposed a functional model of NSSI, known as the Four Factor Model (FFM). FFM is grounded on behavioral theory, which focus on the antecedent and consequent influences that produce and maintain the behavior.

The FFM delineates two dichotomous dimensions of functional processes: contingencies interpersonal/social vs. intrapersonal/automatic, and reinforcement positive vs. negative. The four processes proposed by the model include: automatic negative reinforcement when NSSI serves to reduce aversive internal states, automatic positive reinforcement, when NSSI serves to generate positive feelings, social negative reinforcement, when NSSI serves to avoid interpersonal demands, and social positive reinforcement, when NSSI serves to gain attention, or increase social support (Nock and Prinstein, 2004). Much of the studies on the psychological reasons underlying the NSSI behavior has mainly focused on emotion regulation and tension reduction, but social functions of NSSI have also been described in both adolescents (Nock and Prinstein, 2004; Lloyd-Richardson et al., 2007; Hilt et al., 2008a,b; Turner et al., 2012; Muehlenkamp et al., 2013; Zetterqvist et al., 2013) and adults samples (Turner et al., 2012, 2016). Engaging NSSI “to stop bad feelings” was endorsed by 56.8% of German inpatients sample, in which automatic functions were predicted by adverse childhood experiences (Kaess et al., 2013). Self-punishment function was commonly endorsed by adolescents and adult among community and clinic samples (Briere and Gil, 1998; Laye-Gindhu and Schonert-Reichl, 2005; Lloyd-Richardson et al., 2007; Turner et al., 2012; Glenn and Klonsky, 2013; Kaess et al., 2013). Although males were more likely to engage NSSI for social reasons (Claes et al., 2007) and females to relieve negative emotional states and self-punishment (Briere and Gil, 1998; Glenn and Klonsky, 2013), no significant gender differences emerged about NSSI functions among adolescents samples (e.g., Lloyd-Richardson et al., 2007; Calvete et al., 2015). Zetterqvist et al. (2013) found that about 90% of adolescents those met proposed criteria for NSSI disorder most commonly reported automatic negative functions, followed by automatic positive ones, and about 27% reported social functions. Greater endorsement of intrapersonal functions than social was also found among adult with NSSI Disorder (Andover, 2014).

## Discussion

The purpose of this study was to review the existing literature on NSSI by providing a preliminary understanding of the main features. There is general consensus that NSSI begins in early adolescence, with a main age onset of 12 years old. Even though only few studies have investigated NSSI within children younger than 12 years old (Kirchner et al., 2011; Barrocas et al., 2012; Sornberger et al., 2012). Prevalence is even high among adult, mostly in samples of university students. Studies have demonstrated a high variability on prevalence rates. Some of this variability may partially explain by a growing interest in NSSI behavior. Nevertheless, the assessment methods chosen appears to heavily influence the estimates of prevalence: checklists would seem to provide higher estimates than single item questions (Muehlenkamp et al., 2012).

In order to overcome the mentioned assessment bias, future research will have to accurately investigate perceptions and interpretations of participants which may not reflect NSSI definition provided. NSSI is generally assumed to be more common among females than men. This assumption is not fully supported by existing literature. Self-injury is popularly associated with “cutting” and this could have influenced data, as females are more likely to self-cut than men. Literature has primarily focused on women samples and higher prevalence on NSSI among females could be due to an over representation of women (Claes et al., 2007). Data on non-Caucasian samples are scarce, but it may be due to an ethnocentric bias that tend to underestimate the culture impact on NSSI. Indeed, similar rates of prevalence among female adolescent and methods used, in Chinese samples, could be consider a reflection of Western culture's influence, and NSSI in minorities group, such as Native Americans (e.g., Kuentzel et al., 2012), could be related to culturally sanctioned rituals (ceremonial or religious). Due to paucity of studies on racial/ethnic differences in NSSI, its distribution and prevalence remain unclear.

Regarding etiology, the relation between sexual abuse and NSSI remains still contentious. Evidence suggests that additional risk factors, both environmental and individual, may play a role in the etiology of NSSI: a history of child maltreatment and stressful life experiences could represent a vulnerability that disrupt emotional regulation function. Therefore, several forms of maltreatment appear to be related to engaging NSSI in both clinical and non-clinical samples (e.g., Briere and Gil, 1998; Gratz et al., 2002; Gratz, 2006; Yates et al., 2008; Arens et al., 2012; Auerbach et al., 2014), whereas individual factors might play a role in the maintenance of the behavior (e.g., Gratz and Chapman, 2007; Jacobson et al., 2015). So, a potential interaction between risk factors should be explored. Emotional regulation was the most common reason for NSSI behavior: individuals who self-injury commonly reported negative experiences, such as depression, anxiety, and angry, before NSSI. To further support automatic function, NSSI would result in a negative emotions reduction. Although interpersonal functions have not received as much attention, both adolescents and adult endorsed social reasons to engage NSSI (e.g., Nock and Prinstein, 2004; Lloyd-Richardson et al., 2007; Hilt et al., 2008b; Zetterqvist et al., 2013). Moreover, lower prevalence of social functions could be explained by the fact that NSSI is a private act and who self-injury may be socially isolated and experience negative emotions that increase the likelihood of further acts to reduce tension state. The inclusion of a potential NSSI disorder in the DSM-5 is justified by the clinical benefits that would ensue from a better understanding of the behavior.

Empirical research on NSSI disorder has recently begun to provide relevant data. It is however limited by the use of the different methods employed in assessing NSSI, and not originally envisaged for this purpose. There are several important obstacles regarding diagnostic validity of NSSID. Firstly, delimitation from other disorders. Self-injurious behavior primarily existed in the DSM as a symptom of BPD but, although NSSI and BPD can co-occur, they also present themselves independently (In-Albon et al., 2013). Most studies, focused on NSSI disorder rather than BPD, have highlighted that NSSI is not indicative of BPD and that the diagnostic coincidence of NSSI disorder and BPD was similar to existed to a lesser degree than BPD and other disorders (Glenn and Klonsky, 2013). Moreover, the introduction of NSSI disorder recognizes the importance of differentiating NSSI from attempted suicide. Although both suicide attempts and NSSI conform to a continuum of self-harming behaviors, there are important clinically differences among behaviors in etiology, psychiatric impairment, functions, methods and course. The use of diverse criteria, different assessment methodologies and the absence of studies employing all the criteria as proposed in the DSM-5, have deterred advancement in this field.

### Strengths and limitations

The current review not only includes women engaged in self-injury but also men, and goes some way to addressing the misrepresentation present in previous literature, which could be explained by the fact that men behave differently to women in this context. This study only addresses the links between NSSI and biological roots and developmental\intellectual disabilities in part, and does not address NSSI treatment. Our review attempted to understand the main causes and functions of NSSI through studies on clinical and non-clinical populations but many aspects remain unclear, especially as regard NSSI etiology. Finally, we must consider the secretive nature of self-injury due to which prevalence rates may be seen to be ambiguous. Studies on NSSI treatment were not included.

### Implications for future research

Despite the behavior is more likely to present in adolescence, the variation in age of onset and in prevalence rates among adolescents and adults suggest that there may be different developmental trajectories in NSSI and a lack of knowledge regarding the course of NSSI: further exploration should employ a longitudinal approach aimed at examining the risk factors and progression of a potential NSSI disorder. This implication is directly related to the need for additional research using a variety of adult group to obtain accurate prevalence rates, as data on adult samples have mostly collected in educational institutions.

Findings on gender differences provided contradictory data that could benefit from future research that also consider other variables, such as culture, school, and social contagion. More research would be helpful in understanding the course and patterns of NSSI and exploring NSSI among gender. Research should be extended to other cultures and ethnicities, in order to recognize the influence of cultural factors on these behaviors. Self-injurious behavior for culturally sanctioned purposes (e.g., religious ritual, tattoos and piercings) was not considered in the research field nor included among the proposed diagnostic criteria for NSSID in the DSM-5. The contextualization of behavior is required, as is an exploration of the similarities and differences in prevalence rates, methods and functions across cultures. Although findings suggest the role of abuse, neglect and disruption in attachment in the potential development of NSSI behavior, future research could explore other characteristics of maltreatment experiences, such as frequency, perpetrator, bond type between child, and abuser, and cumulative effects.

There may be several reasons for engaging in NSSI and future research should investigate the mechanism underlying NSSI, the role of gender differences and whether functions change during development in order to a more complete understanding of the behavior. Moreover, there are still several areas that require further investigation to give credence to NSSI as a disorder in its own right: it would be pertinent to provide a valid, clinical delineation of the disorder and develop a standardized tool for its assessment in order to improve research, to conduct longitudinal studies and cross-cultural and ethnic studies, but there is still further work to be done.

## Conclusions

NSSI is a common phenomenon among adolescents and adults, associated with significant impairment. Over the years, interest in NSSI grew to such an extent that an ongoing debate was instigated on whether NSSI should be considered as a diagnosis in its own right and given its own category. As a result, it was included in section 3 of the DSM-5 as a condition requiring further studies. This paper provides an up-to-date overview on self-injury, what is known about it and what remains to be done.

## Author contributions

AC and SC conducted the study, AC writes the first draft of the paper, PC designed the study and supervise the procedure and the paper.

### Conflict of interest statement

The authors declare that the research was conducted in the absence of any commercial or financial relationships that could be construed as a potential conflict of interest.
